# Immunogenicity of a Recombinant Measles-HIV-1 Clade B Candidate Vaccine

**DOI:** 10.1371/journal.pone.0050397

**Published:** 2012-11-30

**Authors:** Richard Stebbings, Michèle Février, Bo Li, Clarisse Lorin, Marguerite Koutsoukos, Edward Mee, Nicola Rose, Joanna Hall, Mark Page, Neil Almond, Gerald Voss, Frédéric Tangy

**Affiliations:** 1 Division of Biotherapeutics, National Institute for Biological Standards and Control, Potters Bar, Hertfordshire, United Kingdom; 2 Unité de Génomique Virale et Vaccination, Institut Pasteur, Paris, France; 3 GlaxoSmithKline Vaccines, Rixensart, Belgium; 4 Division of Virology, National Institute for Biological Standards and Control, Potters Bar, Hertfordshire, United Kingdom; New York University, United States of America

## Abstract

Live attenuated measles virus is one of the most efficient and safest vaccines available, making it an attractive candidate vector for a HIV/AIDS vaccine aimed at eliciting cell-mediated immune responses (CMI). Here we have characterized the potency of CMI responses generated in mice and non-human primates after intramuscular immunisation with a candidate recombinant measles vaccine carrying an HIV-1 insert encoding Clade B Gag, RT and Nef (MV1-F4). Eight Mauritian derived, MHC-typed cynomolgus macaques were immunised with 10^5^ TCID_50_ of MV1-F4, four of which were boosted 28 days later with the same vaccine. F4 and measles virus (MV)-specific cytokine producing T cell responses were detected in 6 and 7 out of 8 vaccinees, respectively. Vaccinees with either M6 or recombinant MHC haplotypes demonstrated the strongest cytokine responses to F4 peptides. Polyfunctional analysis revealed a pattern of TNFα and IL-2 responses by CD4+ T cells and TNFα and IFNγ responses by CD8+ T cells to F4 peptides. HIV-specific CD4+ and CD8+ T cells expressing cytokines waned in peripheral blood lymphocytes by day 84, but CD8+ T cell responses to F4 peptides could still be detected in lymphoid tissues more than 3 months after vaccination. Anti-F4 and anti-MV antibody responses were detected in 6 and 8 out of 8 vaccinees, respectively. Titres of anti-F4 and MV antibodies were boosted in vaccinees that received a second immunisation. MV1-F4 carrying HIV-1 Clade B inserts induces robust boostable immunity in non-human primates. These results support further exploration of the MV1-F4 vector modality in vaccination strategies that may limit HIV-1 infectivity.

## Introduction

Thirty years after human immunodeficiency virus (HIV) was identified as the causative agent of AIDS, a safe and effective vaccine is still urgently required to combat the estimated 2.7 million new HIV/AIDS infections every year [1]–[4]. The first HIV-1 vaccine evaluated in a phase III efficacy trial was based upon recombinant envelope glycoprotein 120 (rgp120) that failed to prevent infection [5]. More recently the RV144 phase III trial, which employed a combination of canarypox vector priming (ALVAC) followed by boosting with a rgp120 vaccine (AIDSVAX), has proven more successful albeit affording only partial protection demonstrated by a 31% reduction in HIV-1 acquisition [6]. Nevertheless, re-examination of the trial data suggests that approximately 70% efficacy may have been achieved during the first year of immunisation, but that this protective capacity declined rapidly after one year [7], [8]. It has been suggested that if an ALVAC/AIDSVAX vaccine was “boostable” then it could be regularly administered in order to maintain high levels of immunity suggested during the first year of immunisation [9]. Unfortunately the blunting effect of anti-vaccine vector immunity caused by previous vaccinations would likely reduce the efficacy of any regular boosting regimen [10], [11]. To overcome this hurdle of anti-vaccine vector immunity the use of alternative serotypes, combinations of different vectors or vectors able to overcome pre-existing immunity needs to be explored [12]–[16].

Live attenuated measles virus (MV) has proven to be one of the safest and most effective human vaccines to date. MV induces life-long immunity after a single or two low-dose injections [17]. Persistence of anti-MV antibodies and CD8+ T cell responses has been shown as long as 25 years after vaccination [18]. The MV genome is very stable and reversion to pathogenicity has never been observed [19]. MV is a negative-stranded RNA virus that replicates exclusively in the cytoplasm, ruling out the possibility of integration into host cell DNA. All these characteristics make live attenuated MV an attractive candidate vaccine vector. To this end, a reverse genetics system for MV has been established [20]–[21], allowing the production of recombinant MV with additional foreign genetic material. Various vectors based on measles vaccine strains have been developed to stably express a variety of genes, or combinations of genes, of large size over more than twelve passages [21]–[26]. These vectors have been shown to induce long-lasting humoral and cellular immune responses to the transgenes, even in presence of pre-existing immunity to MV [16], [22], [25], [27]–[32]. However, an extensive analysis of immune responses elicited in non-human primates has not been performed.

Here, based on the Schwarz measles vaccine strain, we have generated a recombinant measles vector expressing the F4 antigen [27], a fusion protein consisting of HIV-1 Clade B p17, p24, RT and Nef antigens [33]. The immunogenicity of the resulting MV1-F4 candidate vaccine was investigated in mice and cynomolgus macaques. The results presented here show that MV1-F4 vaccination induced both cellular and humoral immune responses against the HIV-1 F4 insert, which were boostable resulting in increased immunogenicity. In addition, long lasting F4-specific CD8^+^ T cell responses were detected in secondary lymphoid organs of vaccinated macaques. These results support the further evaluation of Schwarz MV vector in prime-boost immunisation strategies with the aim of inducing cellular and humoral immunity.

## Results

### Vaccination with MV1-F4 induces strong F4- and MV-specific T cell responses in mice

**Figure 1:**
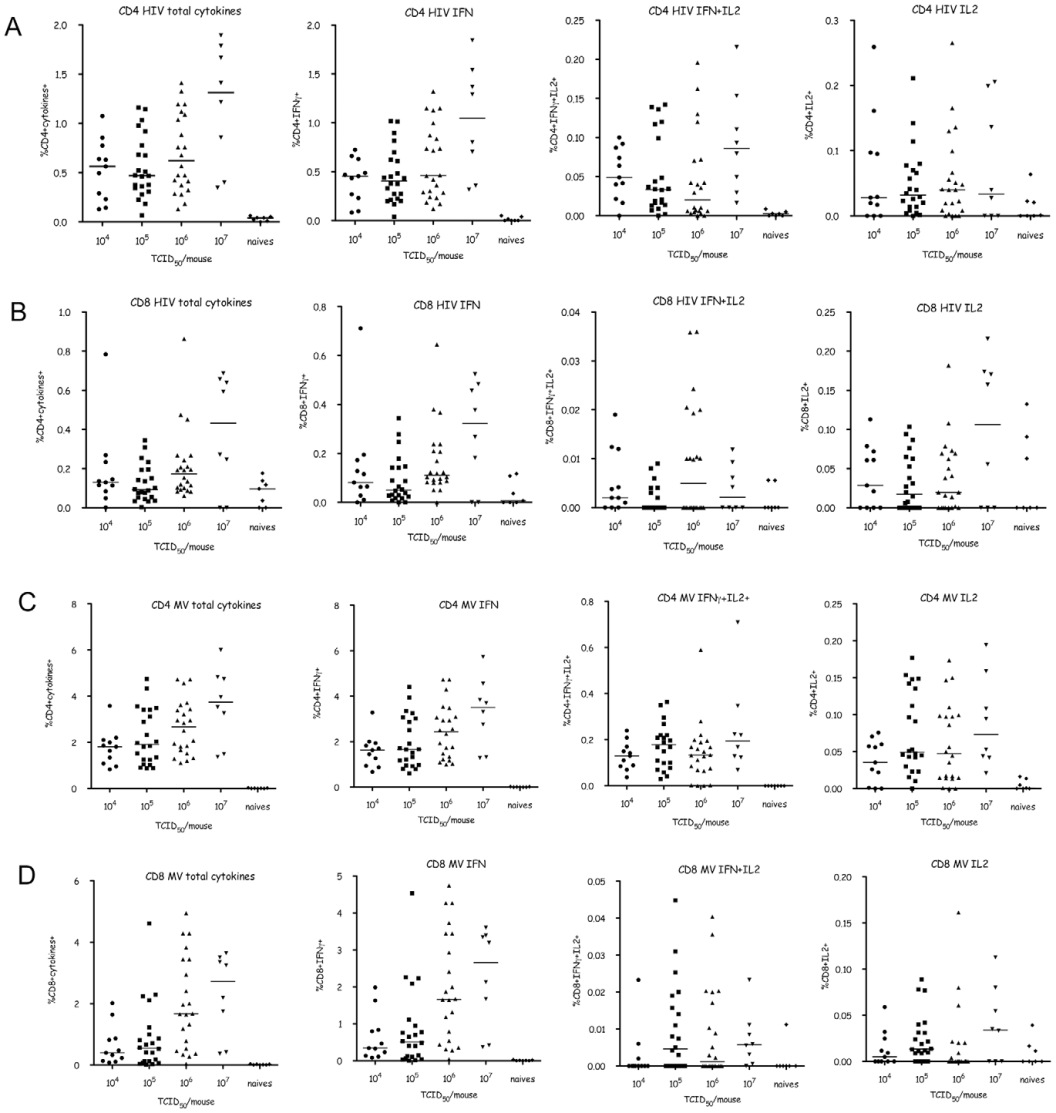
Murine T cell cytokine responses to HIV-1 F4 peptides and MV. Responses of splenocytes from CD46/IFNAR mice immunised with 10^4^–10^7^ TCID_50_ MV1-F4 analysed by flow cytometry for their capacity to secrete IFNγ and IL-2 upon specific stimulation compared to naive controls. IFN + IL-2 indicate a response by double positive cells. Panels A and B show responses to HIV-1 F4 peptide pools by CD4+ and CD8+ T cells, respectively. Panels C and D show responses to MV by CD4+ and CD8+ T cells, respectively.

The immunogenicity of MV1-F4 recombinant vaccine was first evaluated in genetically modified CD46-IFNAR mice susceptible to MV infection. Intracellular cytokine staining was detected by flow cytometry following *in vitro* stimulation of freshly extracted splenocytes with HIV-1 F4 peptide pools (Figure 1A and B) and empty MV (Figure 1C and D). Intracellular cytokine staining for IFNγ and IL-2 was observed in both CD4+ and CD8+ T cells from immunised animals, as compared with non-immunised control mice. The intensity of response, expressed as the percentage of single or double cytokine-positive CD4+ and CD8+ cells, was dependent on the inoculated dose with a marked increase with the highest dose (10^7^ TCID_50_), resulting in strong HIV and MV responses. Single and double cytokine staining for IFNγ and IL-2 was observed in both HIV F4- and MV-specific CD4+ and CD8+ T cells. However, IFNγ was produced in a much higher amount than IL-2. The percentages of CD4+ T cell cytokine responses were at least 2 times higher than CD8+, both for HIV and MV. Altogether, this analysis shows that MV1-F4 is strongly immunogenic and elicits a high level of CD4+ and CD8+ T cell responses in CD46-IFNAR mice, supporting its further evaluation in non-human primates.

### Vaccination with MV1-F4 is well tolerated in macaques

All macaques appeared healthy and active following vaccination with MV1-F4. There were no signs of anorexia, diarrhoea or dermatitis associated with normal measles infection [34]. Full blood cell counts and hematocrit for all animals remained within normal reference range for the duration of the study. The results of biochemistry and liver function tests on days 0, 3, 28 and 31 showed that there was no clinically significant change in levels of albumin, globulin, sodium, potassium, chlorides, urea, creatinine, total bilirubin, alanine transaminase, aspartate transaminase and alkaline phosphatase after vaccination.

### Vaccination with MV1-F4 induces polyfunctional T cell responses to HIV in macaques

**Figure 2:**
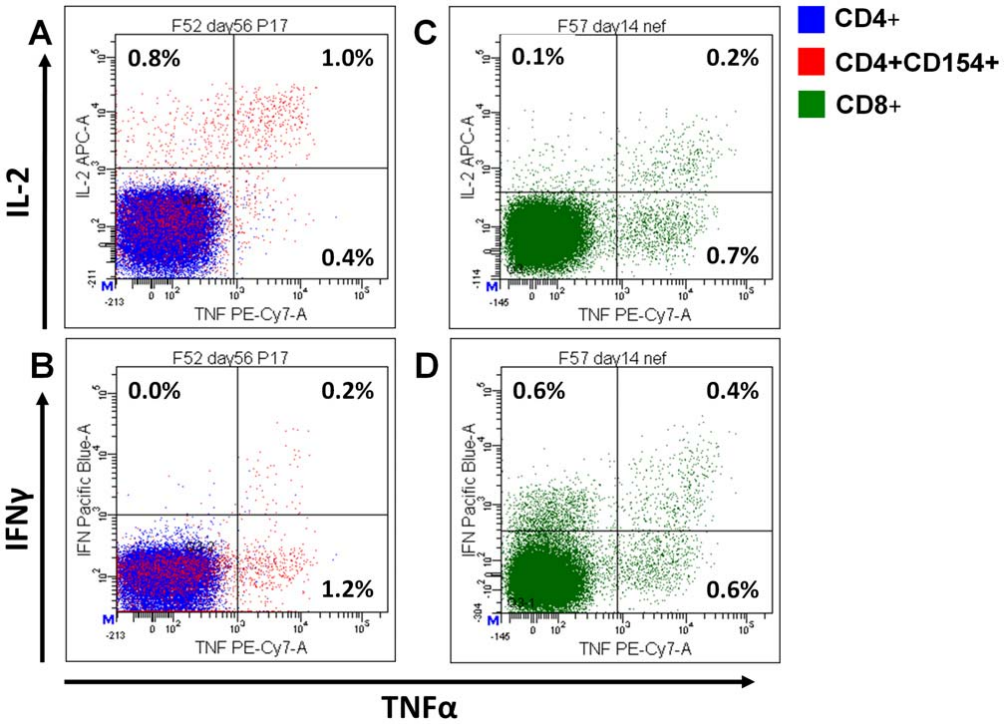
Macaque intracellular cytokine staining. Representative dot plots gated on CD4+ (A and B) and CD8+ (C and D) T cells from MV1-F4 immunised macaques F52 and F57, respectively. PBMC were stimulated overnight with p17 (A and B) or Nef (C and D) peptide pools. Blue events represent CD4+ and red CD4+CD154+ T cells (A and B). Green events represent CD8+ T cells (C and D). Intracellular cytokine staining for TNFα versus IL-2 (A and C) and TNFα versus IFNγ (B and D) is shown. Percentages shown represent the proportion of events in respective quadrants.

Polyfunctional CD4+ and CD8+ T cell cytokine responses were detected by flow cytometry following *in vitro* stimulation of PBMC with HIV-1 F4 peptide pools (Figure 2). Single, dual and triple cytokine staining for TNFα, IL-2 and IFNγ by CD4+ and CD8+ T cells was observed (Figure 2A–D). The activation marker CD154, also known as CD40 ligand, was co-expressed by cytokine positive CD4+ T cells (Figure 2A and B).

**Figure 3:**
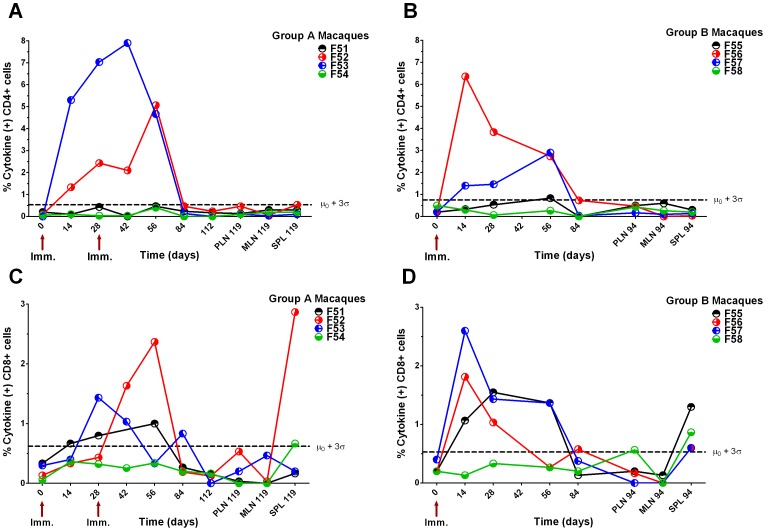
Macaque T cell cytokine responses to HIV-1 F4 peptides. Total cytokine (sum of TNFα+, IL-2+ and IFNγ+ cells) response to F4 peptides by CD4+ and CD8+ T cells from peripheral blood (PBMCs), peripheral lymph nodes (PLN), mesenteric lymph nodes (MLN) and spleen (SPL). Time in days is shown on x-axis, all responses from PBMC unless prefixed by a tissue. Group A, macaques F51–F54, were immunised with 10^5^ TCID_50_ MV1-F4 on days 0 and 28 whilst group B, macaques F55–F58, were immunised on day 0 only. Panels A and B shows cytokine responses gated on CD4+ T cells. Panels C and D show cytokine responses gated on CD8+ T cell. Dashed line represents the mean plus 3 standard deviations of day 0 responses, above which points were deemed significant.

Following vaccination, potent CD4+ T cell cytokine responses against HIV-1 F4 insert peptide pools were detected in macaques F52 and F53 from group A and macaques F56 and F57 from group B (Figure 3A and B). By contrast, macaques F51 and F54 from group A and F55 and F58 from group B did not demonstrate significant CD4+ T cell cytokine responses (Figure 3A and B). The CD4+ T cell response of macaque F55 was ambiguous as it was low and occurred only at a single, late time point (Figure 3B). Only one animal in group A, F52, showed any sign of a boosted CD4+ and CD8+ T cell response against F4 peptides after a second vaccination. The CD4 responses of F53 were already high and continuing to rise at the time of boosting peaking on day 42. Responses in both groups declined sharply at day 84 and could not be detected in lymphoid tissues taken at termination (Figure 3A and B). Significant CD8+ T cell cytokine responses against HIV-1 F4 insert peptide pools were detected in all animals (Figure 3C and D). However, compared with the CD4+ T cell responses against HIV-1 F4 peptides, the magnitude of CD8+ T cell responses was moderate. CD8+ T cell cytokine responses were overall greater and appeared earlier in group B than in group A, but peripheral responses in both groups declined sharply at day 84. Nonetheless, significant HIV-1 F4-specific CD8+ T cell responses could still be detected in spleen cells collected over 3 months after the last immunisation, from all macaques except F51 and F53 (Figure 3C and D).

**Figure 4:**
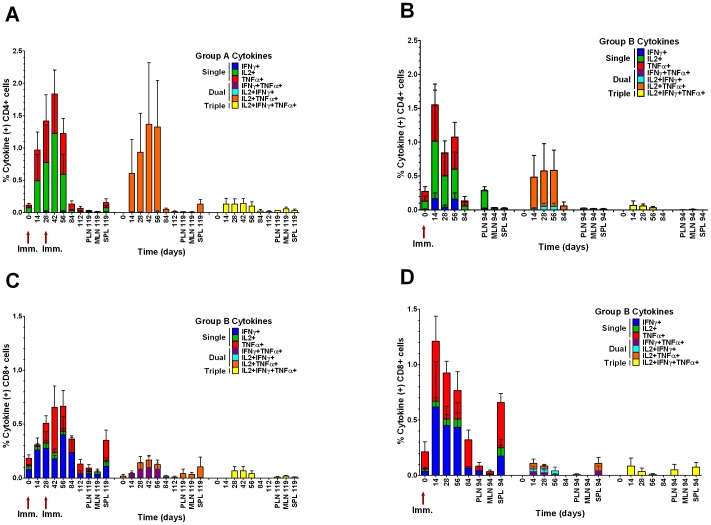
Repertoire of macaque cytokine responses to HIV-1 F4 peptides. Single, double and triple cytokine (TNFα+, IL-2+ and IFNγ+ cells) responses by CD4+ (A and B) and CD8+ T cells (C and D) from peripheral blood (PBMCs), peripheral lymph nodes (PLN), mesenteric lymph nodes (MLN) and spleen (SPL). Group A, macaques F51–F54, were immunised with 10^5^ TCID_50_ MV1-F4 on days 0 and 28 whilst group B, macaques F55–F58, were immunised on day 0 only. Columns represent group mean values ± SEM, n = 4. Single cytokine positive cells represented by IFNγ+ (blue), IL-2+ (green), TNFα+ (red), dual cytokine positive cells represented by IFNγ+ TNFα+ (purple), IL-2+ IFNγ+ (cyan), IL-2+ TNFα+ (orange), and triple cytokine positive cells represented by IL-2+IFNγ+TNFα+ (yellow) are plotted separately.

In groups A and B, CD4+ T cell responses to HIV-1 F4 peptides were mostly composed of single cytokine positive cells, TNFα or IL-2, dual cytokine positive cells, TNFα and IL-2, and a low number of triple cytokine positive cells, TNFα, IL-2 and IFNγ (Figure 4A and B). In contrast, CD8+ T cell responses against F4 peptide mostly comprised single cytokine positive cells, TNFα or IFNγ, a low number of dual cytokine positive cells, TNFα and IFNγ or TNFα and IL-2, and a low number of triple cytokine positive cells, TNFα, IL-2 and IFNγ (Figure 4C and D). Mean CD4+ T cell cytokine responses for group A peaked at day 42 compared to day 14 for group B (Figure 4A and B). Mean CD8+ T cell cytokine responses showed a similar pattern with group A peaking between days 42 and 56 and group B at day 14 (Figure 4C and D).

### Vaccination with MV1-F4 induces humoral responses to HIV in macaques

**Figure 5:**
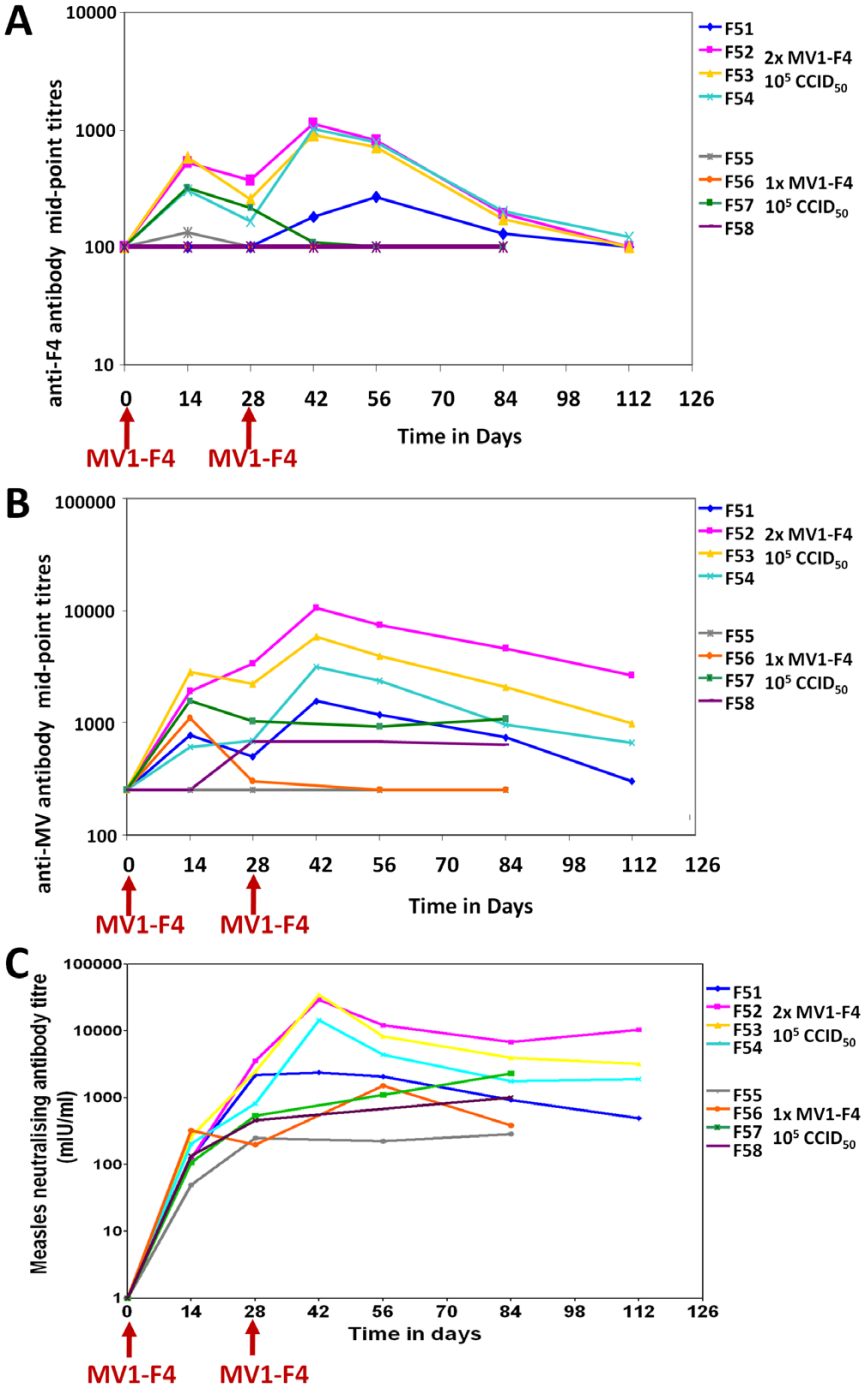
Macaque antibody responses to MV and F4. Anti-F4 (A) and anti-MV (B) antibody responses measured by ELISA with results given as mid-point titres, at which 50% maximal binding is achieved. Measles neutralising antibody titres (C) measured by plaque reduction assay with potency in mIU/ml calculated by direct comparison with the titre of 3rd International Standard for anti-measles serum (97/648). All vaccinees, F51–F58, were immunised with MV1-F4 on day 0, but only F51–F54 received a second immunisation on day 28 (red arrows).

Macaques F51, F52, F53, F54 and F57 developed binding antibodies against HIV-1 F4 antigen as soon as 14 days after immunisation with MV1-F4 (Figure 5A). A very low F4-specific binding antibody response was detected in the F55 animal only at day 14. Seroconversion of macaque F51 was only detected after a second immunisation (Figure 5A). Titres of anti-F4 binding antibodies in macaques F52, F53 and F54 were boosted after a second immunisation, while the anti-F4 humoral responses in the F57 animal waned at day 56. Macaques F56 and F58 did not seroconvert to the F4 antigen (Figure 5A).

### Vaccination with MV1-F4 induces anti-vector responses in macaques

**Figure 6:**
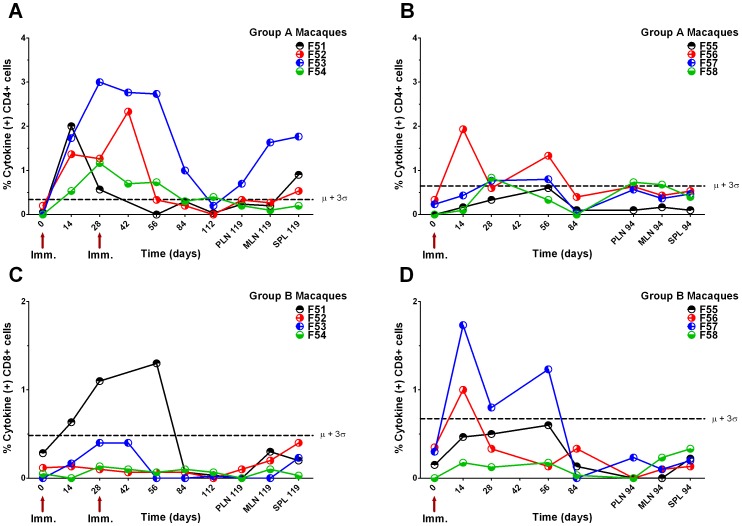
Macaque T cell cytokine responses to empty MV. Total cytokine (sum of TNFα+, IL-2+ and IFNγ+ cells) response to MV by CD4+ and CD8+ T cells from peripheral blood (PBMCs), peripheral lymph nodes (PLN), mesenteric lymph nodes (MLN) and spleen (SPL). Time in days is shown on x-axis, all responses from PBMC unless prefixed by a tissue. Group A, macaques F51–F54, were immunised with 10^5^ TCID_50_ MV1-F4 on days 0 and 28 whilst group B, macaques F55–F58, were immunised on day 0 only. Panels A and B shows cytokine responses gated on CD4+ T cells. Panels C and D show cytokine responses gated on CD8+ T cell. Dashed line represents the mean plus 3 standard deviations of day 0 responses, above which points were deemed significant.

All macaques except F55 seroconverted to MV following immunisation and titres were boosted in all of group A macaques following a second immunisation (Figure 5B). At termination, anti-MV neutralising antibodies were detected in all vaccinees with protective titres >500 mIU/ml [35] in all animals except F51 (Figure 5C). Significant CD4+ T cell cytokine responses against MV were detected in all group A and B macaques, except F55 (Figure 6A and B). Overall CD4+ T cell responses against MV were greater in group A than B, declined sharply at day 84 in both groups but significant responses were still detected in the lymphoid tissues of F51, F52, F53 and F58 at termination (Figure 6A and B). Significant CD8+ T cell cytokine responses against MV were only detected in F51, F56 and F57 but no responses were detected in lymphoid tissues taken at termination (Figure 6C and D).

### Recombinant MHC haplotypes correlate with superior CMI responses

A retrospective analysis of macaque MHC haplotypes was undertaken in order to better understand differences in CMI responses between vaccinees. Macaques F51, F54, F55 and F57 were heterozygotes (M1/M3, M2/M3, M1/M3 and M3/M6, respectively; Table 1). Macaque F58 was the only homozygote (M3/M3). Macaques F52, F53 and F56 possessed heterozygous recombinant MHC haplotypes (M3/M1+M2+M3, M2/M1+M2+M7 and M1/M1+ M3, respectively). All three macaques with a simple recombinant MHC haplotype made significant CD4+ and CD8+ T cell cytokine responses against HIV-1 F4 peptides and MV. By comparison, only 1 out of 5 vaccinees that possessed a simple heterozygous or homozygous MHC haplotype made significant CD4+ and CD8+ T cell cytokine responses against HIV-1 F4 peptides.

**Table 1:** Macaque MHC haplotype analysis.

	Animal	Class IA	Class IB	Class II
**Group A**	F51	M1	M1	M1
		M3	M3	M3
	F52	M3	M3	M3
		M1	M3	M3/M2
	F53	M2	M2	M2
		M7	M2	M1/M2
	F54	M2	M2	M2
		M3	M3	M3
**Group B**	F55	M1	M1	M1
		M3	M3	M3
	F56	M1	M1	M1
		M1	M1/M3	M3
	F57	M3	M3	M3
		M6	M6	M6
	F58	M3	M3	M3
		M3	M3	M3

MHC haplotypes of MV1-F4 vaccinees, F51–F58, were determined by microsatellite analysis and haplotypes with recombination were resolved by allele-specific PCR. Intact haplotypes, M1–M7, have been previously identified in Mauritian cynomolgus macaques [62], [63].

## Discussion

In countries where HIV is highly prevalent, a recombinant MV-HIV vaccine might be administered to naïve infants as a standard measles immunization that would protect from measles whilst eliciting long-term memory to HIV that could be boosted later with another type of HIV vaccine. A MV-HIV recombinant vaccine might also be used to immunize the adolescent and adult populations who are already pre-immune to MV since their childhood vaccination. In that case, pre-existing immunity to measles might prevent or reduce the efficacy of the recombinant MV vaccine. However, numerous studies have shown that revaccinating already immunized individuals results in a boost of anti-MV immunity, indicating that the live vaccine replicates in spite of pre-existing immunity [36], [37]. Moreover, we previously demonstrated that a recombinant MV-HIV vector induced antibodies to HIV in mice and macaques in the presence of MV pre-existing immunity, provided that two injections with a higher dose are performed [38]. Yet, this point needs to be further evaluated in human trials. Measles is still difficult to control, as evidenced by the large outbreaks occurring in Europe since 2010. Improving measles vaccination coverage is essential to containing and preventing further such outbreaks. An MV-HIV vector might be an effective and safe carrier for a HIV vaccine, whilst boosting pre-existing measles immunity.

This study was undertaken to evaluate the immunogenicity of a recombinant measles vector, MV1-F4, carrying an insert encoding HIV-1 clade B gag, RT and nef. CD46/IFNAR mice and cynomolgus macaques were chosen for pre-clinical evaluation of MV1-F4, as both species are susceptible to infection with MV vaccine strains and further macaques are susceptible to infection with wild-type MV [39]. As a first and rapid assay, mice were inoculated with a single administration of escalating doses of recombinant vaccine and CMI were assessed as early as 7 days after immunization. MV1-F4 vaccine was immunogenic and induced strong CD4+ and CD8+ responses to HIV-1 F4 and to MV. CD4+ responses that released mainly IFNγ were observed, although the cytokine pattern in mice that lack type-I IFN receptor is likely unconventional. Dose-response effect was evident, the higher doses being more immunogenic. A relatively low dose (10^5^ TCID_50_) was chosen to immunise macaques. To determine whether boosting with this vaccine improves immunogenicity, we compared responses in groups of macaques that received a single immunisation with those that received two. Superior immunogenicity was obtained with two immunisations, indicating that MV1-F4 humoral responses are boostable even in the presence of anti-vector antibodies. Titres of anti-F4 and anti-MV antibodies initially peaked at day 14 but were boosted after a second immunisation at day 28. By contrast, primary CD4+ T cell cytokine responses against MV1-F4 initially peaked between days 14 and 28 in vaccinees that responded, but boosting was only observed with 1 macaque out of 4, F52. From these data, it would appear that a boost interval of 28 days is too short for efficient re-stimulation of CD4+ T cell responses. For example, macaque F53 appears to have been boosted during its primary response suggesting that the peak reached on day 42 was due to the primary vaccination. Importantly the CD8 responses of F53 fell after boosting. Since peripheral CMI responses against MV1-F4 waned by day 84, in both groups of macaques, then, in hindsight, delaying the second vaccination to this later time may have resulted in superior boosting. This may explain why boosting of humoral responses contrasted with a lack of boosting of cellular responses. For example, macaque F54 was divergent in that it had had good anti-F4 and MV antibody responses that were boosted, but in comparison poor CMI responses. Alternatively, measurement of Th2 cytokines (IL-4, IL-5 and IL-13) may have better correlated with boosting of humoral responses. These results may guide further preclinical and clinical development of the MV1-F4 candidate vaccine.

In vitro responses to measles are dominated by CD4+ T cells that, depending on antigen dose, primarily produce a Th1-like pattern of cytokine release [40]. Therefore it was not unexpected for CD4+ T cell cytokine responses to MV1-F4 to be greater than CD8+ T cell responses, following F4 peptide stimulation. Interestingly, responding CD4+ T cells were all CD154+, which acts as a potent maturation agent for dendritic cell priming of anti-viral CD8+ T cells, desirable for a candidate HIV vaccine [41]. Although peripheral CMI responses were no longer readily detectable by day 84, CD8+ T cell responses could still be detected in lymphoid tissues taken at termination suggesting that MV1-F4 may stimulate long lasting immunity. This would be consistent with previous reports that MV-specific CD4+ and CD8+ T cells and MV-specific IgG can be detected up to 25 years after vaccination [18], [42].

The cytokine profiles observed with MV1-F4 vaccinees were remarkably similar between responding animals. For CD4+ T cells, we observed a TNFα and IL-2 bias that contrasted with CD8+ T cell responses with a TNFα and IFNγ bias. Such responses are indicative of a T helper 1 (Th1) cell response characterized by the production of IFN-γ, IL-2 and TNF-α [43]. However, IL-4 responses were not assessed here so a Th1/Th2 mixed pattern of cytokine release cannot be excluded. The bias of CD4+ T cell cytokine responses to TNFα and IL-2 suggests a central memory response rather than terminally differentiated CD4 effector cell response, which was characterised by higher levels of IFN-γ and TNF-α expression [44]. CD8+ memory T cells can quickly produce a variety of cytokines including IFNγ, TNFα and to a lesser extent IL-2 [45], matching the profile we observed against F4 peptides. The low frequency of multifunctional T cells, positive for all three cytokines, is likely due to the necessary use of peripheral blood lymphocytes deficient in effector memory T cells when compared with mucosal sites [46], [47].

Despite group B receiving only a single MV1-F4 immunisation their CD8+ T cell responses were of a greater magnitude than group A that received two immunisations. This may have been the result of MHC haplotype bias of individuals within group B towards strong CD8+ T cell responses, which may have been more evident due to our small group size (n = 4). Theoretically such variability could be minimised with larger group sizes or pre-selection of MHC matched animals to balance MHC-restricted CMI responses between groups. In a follow up study, using MV1-F4 encoding a C Clade HIV-1 insert, we plan to test this using larger groups (n = 8) of MHC-characterised cynomolgus macaques assigned to each group evenly, rather than randomly assigning animals as previously done.

In SIV infection, Mauritian derived cynomolgus macaques with the rare M6 MHC haplotype (∼4% of the population) are associated with a significant reduction in chronic phase viremia [48], [49]. If control of viremia is associated with superior MHC-restricted CMI responses then it might be expected that those individuals would also make superior vaccine responses. In this study, the only macaque with a M6 MHC haplotype was F57, a M3/M6 heterozygote, which coincidentally exhibited the best CD8+ T cell response and a strong CD4+ T cell response to both HIV-1 F4 and MV. However, the M2 MHC haplotype has also been associated with significant control of chronic phase SIV viremia [49], yet we failed to detect significant CD4+ or CD8+ T cell cytokine responses against HIV-1 F4 peptides in the M2/M3 heterozygote F54, despite two immunisations. At a simpler level, chronic SIV viremia in MHC homozygous macaques is reported to be 80 times worse than in MHC heterozygous Mauritian-derived cynomolgus macaques [50]. MHC heterozygous advantage suggests recognition of a maximally diverse set of epitopes that provides a rationale for prophylactic vaccination to elicit broad CMI responses in such individuals [51]. The only MHC homozygote in this study was F58, a M3/M3 homozygote, which had poor CMI responses to HIV-1 F4 peptides. However, there was no evidence of a heterozygous advantage for F51 and F54, M1/M3 and M2/M3 MHC haplotypes respectively, in terms of superior CMI responses to F4 peptides. More interestingly, all macaques with recombinant heterozygous MHC haplotypes, F52, F53 and F56, M3/M1+M2+M3, M2/M1+M2+M7 and M1/M1+M3 respectively, made significant CMI responses to F4 peptides, even though they are simple recombinants of the same alleles present in F51 and F54. Recombination between alleles and loci has been suggested as a mechanism responsible for generating diversity at MHC loci allowing recognition of new epitopes [52]. Our data suggests that heterozygous recombinants have an advantage in terms of responses to vaccines, with the caveat that our group size and number of haplotypes is small. Nevertheless this is an issue that should be taken into account when assigning macaques between groups to avoid bias.

Immune escape is believed to be a significant force shaping viral evolution at the population level through a MHC “imprinting effect” in which escape mutations selected in the context of common MHC alleles may become predominant in the circulating viral population, unless they revert when transmitted to new hosts [53], [54]. If immunodeficiency viruses have evolved to escape commonly presented epitopes, then MHC homozygous and common heterozygous haplotypes may be at a disadvantage in terms of poor vaccine responses compared to recombinant or rare haplotypes. Mauritian derived cynomolgus macaques however, have not to our knowledge ever been naturally exposed to SIV and hence the virus won't have adapted to their MHC. In contrast, heterozygote advantage is probably due to twice as many alleles meaning twice as many potential T cell responses. As a result macaques with homozygous MHC haplotypes would be anticipated to be poor vaccine responders and so they should be equally divided between vaccine groups to avoid negative bias. By chance, F51 in group A and F55 in group B shared the same MHC heterozygous haplotype, M1/M3. However, although their CMI responses exhibited similarities, they were not identical, thus suggesting that other factors, possibly minor histocompatibility antigens also shape immune responses to MV1-F4 [55], [56]. The main limitation of this study was the small group size complicated by a mix of responders and non-responders to the F4 insert. Retrospective analysis of MHC haplotypes greatly aided our interpretation of an otherwise potentially confusing pattern of vaccine responses. In future MHC typing of Mauritian derived cynomolgus macaque studies has the potential to greatly increase the power of new studies and reduce animal usage.

All vaccinees developed anti-MV neutralising antibodies after immunisation but this could have been induced by inert virus particles. Only humoral and CMI responses to MV1-F4 demonstrate replication of the vector because the vaccine preparation contained only MV particles and no F4 protein. Thus, F4 protein was expressed from vector replication *in vivo*. By these criteria there may have been no take of MV1-F4 in F58. It is unclear why F55 failed to seroconvert to MV-ELISA antigen despite a detectable neutralising antibody titre, but it may be a technical or species issue associated with the use of recombinant proteins. Since most HIV vaccine candidates based on vectors are derived from naturally occurring human viruses, pre-existing immunity has the potential to blunt vaccine responses through neutralisation [57], [58]. However, boosting of humoral immunity with MV1-F4 was able to efficiently overcome pre-existing immunity in the presence of protective tires of measles neutralising antibody. It is possible that the intramuscular route of immunisation prevented rapid neutralisation of MV1-F4 allowing boosting to occur. This may have facilitated secretion of transgene proteins that could boost humoral immunity. Secretion of HIV proteins by infected muscle would require cross presentation by antigen presenting cells to boost CD8 responses, a process that is probably is probably less efficient in directing antigen to MHC class I than de novo synthesis. Alternatively, the aerosol route of administration is very effective in humans as a booster for the second MV immunization [38]. In a recent study that also used a vector based upon an attenuated measles virus strain, expressing SIV Gag, transgene immunity was found to be weak, but immunisation was carried out in pre-immune rhesus macaques, albeit in the absence of protective titres of measles neutralising antibodies [59]. That pre-existing immunity may have limited replication of attenuated vaccine virus resulting in low levels of transgene expression and immunogenicity. By contrast, immunisation of naive cynomolgus macaques with MV1-F4 in this study resulted in superior transgene and anti-vector responses.

A replicating vaccine vector capable of inducing potent cellular and humoral immune responses is likely to be required for the development of an effective HIV vaccine. That vector will also need to be safe and preferably boostable. The results of this study suggest that MV1-F4 is a promising component of a prime-boost vaccine strategy to limit the spread of HIV-1.

## Materials and Methods

### Ethics statement

Mice were housed under specific pathogen-free conditions at the Pasteur Institute animal facility and all experiments were approved and conducted in accordance with the guidelines of the Office of Laboratory Animal Care at Pasteur Institute. Macaques in this study were used in strict accordance with UK Home Office guidelines. The work at NIBSC was governed by the Animals (Scientific Procedures) Act 1986 which complies with the EC Directive 86/609. The work was performed under licence PPL 80/1952 which was granted only after review of all the procedures in the licence by the local Ethical Review Process. All individuals in the study were purpose-bred and group housed for the entire duration of the study. Regular modifications to the housing area including the introduction of novel structures and the introduction of foodstuffs in novel manners were made by husbandry staff to enrich the environment during the study. Regular, frequent checks were made by staff and any unexpected changes in behaviour by individuals on study were followed up, including seeking of veterinary advice where necessary. Regular blood samples were obtained to assess cellular and humoral immune responses and to monitor health parameters (haematology, biochemistry and liver function). All macaques were sedated with ketamine hydrochloride before vaccination or venepuncture and killed humanely at end of study by an overdose of anaesthetic.

### Plasmid construction

**Figure 7:**
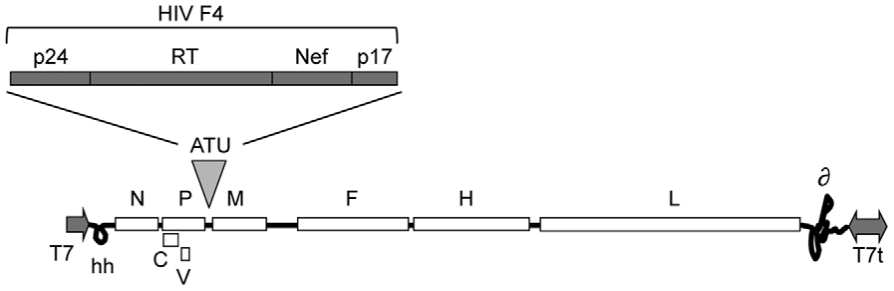
MV vector with ATU in positions 2 and 3. MV genes indicated are: N, nucleoprotein); PVC, phoshoprotein and V/C accessory proteins; M, matrix; F, fusion; H, hemagglutinin; L, polymerase; T7, T7 RNA polymerase promoter; hh, hammerhead ribosyme; T7t = T7 RNA polymerase terminator.

The plasmid pTM-MVSchw carries an infectious cDNA corresponding to the anti-genome of the Schwarz MV vaccine strain [29]. An additional transcription unit (ATU) has been inserted into the plasmid backbone by site-directed mutagenesis between the MV P and M genes. Each MV open reading frame (ORF) expression is controlled by its own cis-acting element. The expression of additional ORFs inserted in the ATU is controlled by cis-acting elements modelled after those present in the N/P boundary region (allowing for the necessary transient transcription stop upstream of the transgene, autonomous transcription, capping and polyadenylation of the transgene). The HIV-1 F4 fusion protein sequence has been sub-cloned in the ATU resulting in the plasmid pTM-MVSchw-ATU2_F4co_mut (Figure 7). The F4 antigen has been described previously [60] and comprises HIV-1 subtype B antigens p24 (BH10), RT (HXB2), Nef (Bru-Lai) and p17 (BH10). The corresponding MV1-F4 virus was rescued from the pTM-MVSchw-ATU2_F4co_mut plasmid using a helper cell-based system. Briefly, helper HEK293 cells expressing both the T7-RNA polymerase and the Schwarz MV N and P proteins (HEK293-T7-MV) were co-transfected with the pTM-MVSchw-ATU2_F4co_mut cDNA and a plasmid expressing the Schwarz MV polymerase L. Subsequently, transfected HEK293-T7-MV helper cells were gently harvested and co-cultured with MRC-5 cells for the amplification of the MV1-F4 virus. Virus titres were determined by endpoint titration on Vero cells and expressed as TCID_50_/ml.

### Study designs and animals

#### Mice

CD46-IFNAR mice susceptible to MV infection were produced as previously described [29]. These mice express hCD46, the human receptor for vaccine MV strains, and lack the IFN-α/β receptor [33]. They have been used previously as a model to evaluate the immunogenicity of recombinant MV [16], [22], [25], [27]–[32]. Groups of eight week old CD46-IFNAR mice were inoculated intraperitoneally (i.p) with increasing doses of MV1-F4 recombinant virus (10^4^ to 10^7^ TCID_50_). Non-immunized mice were used as controls. To assess the early cell-mediated immune responses elicited against both the HIV-1 F4 antigen and the MV vector, mice were euthanized at 7 days post-immunization, spleens were collected and splenocytes purified.

#### Macaques

Eight D-type-retrovirus-free juvenile male cynomolgus macaques (*Macaca fascicularis*), were divided equally into two groups. Group A, macaques F51–F54, and Group B, macaques F55–F58, were vaccinated i.m. with 10^5^ TCID_50_ MV1-F4 on day 0. Only group A received a boost on day 28 with the same vaccine. PBMCs were isolated by Percoll™ density gradient centrifugation (Sigma) as previously described [61] and cryopreserved for later analysis. Serum and plasma were collected and stored at −20°C for later analysis. Spleen, peripheral and mesenteric lymph nodes were collected at termination, cells isolated by mechanical tissue disaggregation (Medimachine, BD Bioscience) and cryopreserved for later analysis.

### Cellular immune responses

#### Mice

Freshly extracted splenocytes from immunized CD46/IFNAR mice were analyzed by flow cytometry for their capacity to secrete IFNγ and IL-2 upon specific stimulation. Spleen cells were cultured for 6 hours in U-bottom 96-well plates (1.0×10^6^ cells/well) in a volume of 0.2 ml complete medium (RPMI 1640/glutamax medium supplemented with 5% fetal calf serum, 50 mM 2-mercapto-ethanol, non-essential amino acids, sodium pyruvate and antibiotics). Cells were stimulated in triplicate with clinical trial stocks of synthetic peptide pools covering HIV-1 clade B F4 (p24, p17, RT and Nef 15-mers with an 11 amino acid overlap) at 1 µg/ml/peptide (stock solutions dissolved in DMSO, endotoxin levels <0.3 IU/ml, p17 pool of 31, p24 pool of 55, RT pool of 138 and Nef pool of 49 peptides) or a live attenuated empty MV (10^6^ TCID_50_/10^6^ cells). Negative controls were incubated with an equal volume of DMSO (0.1% v/v) without peptide and positive controls with 1 µg/ml Concanavalin A (Sigma). Brefeldin A (Sigma) was then added at 10 µg/ml for overnight incubation. Stimulated cells were harvested, washed in phosphate-buffered saline containing 1% bovine serum albumin and 0.1% w/w sodium azide (FACS buffer), incubated 10 min with Fc blocking Ab (CD16/32 clone 2.4G2, PharMingen) and surface stained in FACS buffer with anti-mouse CD4-PE mAb (clone RM4-5, PharMingen) and anti-mouse CD8-PerCP mAb (clone 53-6.7, PharMingen) for 30 min at 4°C in the dark. After washing, cells were fixed and permeabilised for intracellular cytokine staining using the Cytofix/Cytoperm kit (BD Bioscience). Cells were then incubated in a mix of anti-mouse IFNγ-APC mAb (clone XMG1.2, PharMingen) and anti IL-2-FITC mAb (clone JES6-5H4, PharMingen) diluted in permwash buffer (BD Bioscience) for 45 min in the dark. After washing with permwash buffer and FACS buffer, cells were fixed with 1% formaldehyde in PBS. At least 20,000 splenocytes events in CD8 gate were acquired per well using a FACSCalibur flow cytometer (Becton Dickinson). Data were analysed using CELLQuest software (Becton Dickinson) and are presented as % of CD4 or CD8 cells expressing IL-2 or IFNγ among total CD4 or CD8 populations.

#### Macaques

Polyfunctional flow cytometry was performed to detect cytokine responses following stimulation of lymphocytes from cynomolgus macaques with synthetic peptide pools covering the HIV-1 F4 insert. Cryopreserved macaque cells were thawed, washed and stimulated overnight in 96 well round bottom tissue culture plates (Jencons) at 37°C under 5% CO_2_ in RPMI 1640 culture medium (Sigma) supplemented with L-glutamine, penicillin, streptomycin and 10% foetal calf serum (Gibco). Macaque PBMC (2.0×10^5^ cells/well) were stimulated in triplicate with clinical trial stocks of synthetic 15 mer peptide pools covering HIV-1 clade B p24, p17, RT and Nef sequences at 1 µg/ml/peptide (stock solutions dissolved in DMSO, endotoxin levels <0.3 IU/ml) or a live attenuated empty MV at 10^4^ TCID_50_/10^6^ cells in the presence of 1 µg/ml of the co-stimulatory antibodies anti-CD28 and CD49d (BD Bioscience). Negative controls were incubated with an equal volume of DMSO (0.1% v/v) without peptide and positive controls with 1 µg/ml SEB (Sigma). After 2 h Brefeldin A (Sigma) was added to wells to give a final concentration of 10 µg/ml. Stimulated cells were surface stained for 20 min with anti-CD3 FITC (AbD Serotec), anti-CD4 APC-Cy7 (Biolegend) and anti-CD8 AmCyan (BD Bioscience), washed twice with PBS supplemented with 5% foetal calf serum and 0.1% w/v Sodium Azide, fixed for 20 min at room temperature in Fix & Perm® reagent A (Invitrogen), washed twice and incubated with Fix & Perm® reagent B (Invitrogen) containing anti-TNFα PE-Cy7 (Biolegend), anti-IFNγ Pacific Blue (Biolegend), anti-IL-2 APC (Biolegend) and anti-CD154 PE (Biolegend) for 30 min at room temperature, washed twice and re-suspended in PBS containing 2% formaldehyde (Fisher Scientific). Consistency of staining and analysis across plates was assessed with lyophilised cell controls for intracellular cytokine staining, ARP5019 and ARP5020 (NIBSC). Acquisition was performed using a BD FACSCanto II cytometer equipped with a HTS plate reader and data was analysed with BD FACSDiva software (BD Bioscience). Cytometer setup and tracking beads (BD Biosciences) were run prior to acquisition to ensure optimal linearity and sensitivity. At least 100,000 lymphocyte events were collected per a well for analysis.

### Humoral Immune Responses

The presence and magnitude of F4-specific binding antibodies in monkey plasma samples were determined using an enzyme-linked immunosorbent assay (ELISA). F4 antigen at 0.25 µg/ml in PBS was coated overnight at 4°C onto 96-well plates and then blocked for 1 h at 37°C with a saturation buffer (PBS, 0.1% Tween 20, 1% BSA, 4% NCS). Plasma samples from vaccinated macaques were serially diluted (twelve dilutions) in the saturation buffer and incubated on plates for 1.5 h at 37°C. After washing steps (0.1% Tween in PBS), a secondary horseradish peroxidase conjugated goat anti-monkey Ig antibody (Rockland) was added at a dilution of 1/1000 for 1 h at 37°C. Antibody binding was revealed by addition of the TMB substrate (Biorad) and the reaction was stopped by addition of H_2_SO_4_ 1M. The Optical densities (O.D.) were recorded at 450 nm (Emax microplate reader, Molecular Devices). Mid-point titres were calculated as the reciprocal dilution for which 50% maximal binding was achieved, using SoftmaxPro v3.1 software. For non-responding animals, an arbitrary value corresponding to half of the lowest dilution used for the serial plasma dilutions was attributed (titre of 100).

MV-specific Ig antibodies were measured in the monkey plasma samples using a commercial enzyme immunoassay, Enzygnost® Anti-Measles Virus/IgG (Siemens). The original supplier's instructions have been modified in order to increase the sensitivity of the assay for the detection of anti-MV antibodies in non-human primates: Eight serial plasma dilutions were added to the plates for 1 h at 37°C and the horseradish peroxidase conjugated goat anti-monkey Ig antibody (Rockland) was used as a secondary antibody at a dilution of 1/6000 (1 h at 37°C). The signal was revealed by addition of the TMB substrate and Mid-point titres were calculated as the reciprocal dilution for which 50% maximal binding was achieved, using SoftmaxPro v3.1 software. For non-responding animals, an arbitrary value corresponding to half of the lowest dilution used for the serial plasma dilutions was attributed (titre of 250).

Measles neutralising antibody responses were detected using a plaque reduction assay. Briefly, dilutions of serum were reacted with a standard inoculum of wild type measles for 90 min, added to 24 well tissue culture plates containing a suspension of Vero cells in MEM culture medium supplemented with foetal calf serum, L-glutamine, Penicillin/Streptomycin, Amphotericin B (Gibco) and incubated for 2–3 h at 35°C under 5% CO_2_. Culture medium was carefully replaced with fresh medium and plates incubated for a further 7 days under the same conditions. Cell monolayers were then fixed and stained with methyl violet (Sigma). The dilution of serum reducing the number of plaques by 50%, determined using the Spearman-Karber formula, was taken as the end-point titre. Potency in mIU/ml was calculated by direct comparison with the titre of 3^rd^ International Standard for anti-measles serum (97/648).

### MHC analysis

Seven common haplotypes (designated M1–M7) account for 99% of MHC class I and II diversity in Mauritian derived Cynomolgus macaques [62], [63]. This restricted MHC diversity of Mauritian-derived cynomolgus macaques makes it possible to relate CMI responses to haplotype [62], [63]. MHC haplotypes of macaques were determined by microsatellite PCR as described previously [63]. Where recombination was observed allele-specific, PCR was employed to resolve individual alleles carried by each animal.

### Statistical analysis

Polychromatic staining for TNFα, IFNγ and IL-2 assessed as single, dual or triple cytokine positive cells were summed to provide a total cytokine response. Cytokine responses against 4 peptide pools, p17, p24, RT and Nef were summed to give a total response against the HIV-1 F4 insert. Background responses against DMSO controls were subtracted from individual peptide pool result before summation. Cytokine responses for CD4+ and CD8+ T cell were calculated separately. Cytokine responses greater than the mean plus 3 standard deviations of day 0 responses were deemed significant. All statistical analysis was performed using Prism 5 software (Graph Pad Software).
